# The Road to Hell Winds On: The High Administrative Burden of Maintaining Linked National Health Data

**DOI:** 10.23889/ijpds.v11i1.3000

**Published:** 2026-04-30

**Authors:** Julie A. Taylor, Christina Pagel, Ferran Espuny Pujol, Sonya Crowe

**Affiliations:** 1 Clinical Operational Research Unit, Department of Mathematics, University College London, 4 Taviton Street, London, WC1H 0BT, UK; 2 Department of Computer Science, University of Reading, Pepper Lane, Reading RG6 6DH, UK

**Keywords:** data linkage, audit, public health, health services research

## Abstract

**Introduction:**

Data linkage studies generate opportunities to answer important health questions, but are also notoriously time consuming, with delays accessing data lasting years. Previously we described our experience of accessing and linking five national datasets for our study ‘LAUNCHES QI’. However, the delays and challenges we encountered did not end once data were received - maintaining access and research amendments also required significant time and resources.

**Objective:**

To outline the costs, time, and workload associated with LAUNCHES, including set-up, annual requirements, and amendments. We also describe the experience of re-using the linked dataset for a second study, CHAMPION, which we wrongly assumed would be simpler. We highlight new delays and challenges, offer advice to researchers, and suggest improvements to the system.

**Results:**

LAUNCHES set-up involved 39 documents and data access took 2.5 years. Subsequent amendments/renewals added 75 more documents. Repurposing the data for CHAMPION required restarting the entire process from scratch. It took nearly two years to gain initial approvals and another 18 months to receive the datasets, with 44 documents submitted for set-up and 92 more for amendments and annual processes.

New challenges and delays included changes of data controller, data extract errors, and the repetitive amendment process.

**Conclusions:**

With growing recognition of the challenges in using health data for research and our own increasing experience navigating the system, we had hoped for positive change. Instead, there are more forms, and processes have become harder, not easier. We believe simple changes could streamline the system, yet with each new study, the document set grows, and the forms get longer. Research with linked datasets is incredibly painful, and the system discourages amendments, stifling innovation. Whilst we’ve realised the value of linked data, delays to research are significant, and researchers may give up without simpler processes to link and update data.

## The Unfulfilled Potential

The opportunity generated by data linkage studies using health data, the time and workload involved, and the challenges encountered are being increasingly reported [1–28] In some cases, publicly funded studies that have invested substantial time, funding, and resources in navigating approvals and data application processes are ultimately unable to obtain all of the requested data [19, 29]. There are ongoing efforts to improve the system, including a government commissioned review on using health data for research and analysis [30] and an independent review of the UK health data landscape [31].

As researchers attempt to answer important health questions by setting up larger data assets with linked datasets [32], the barriers and amount of time it takes is delaying research progress. Solutions such as using simulations [33], self-report for health outcomes [34, 35], synthetic data [36, 37] or specific trusted research environments (TREs) have been explored [38–40]. A TRE is a highly secure computing environment that provides remote access to health data for approved researchers to use in research. These ‘data safe havens’ are being established by custodians to hold several de-identified datasets with a process to allow approved external researchers to analyse the data. However, they may not always hold the right datasets to address specific research questions (e.g. a specific audit). When they do have the required datasets, the data can only be processed for pre-specified research questions. Therefore, many studies still require the use of bespoke linked datasets [41, 42].

In a seemingly positive move, the government has recently announced plans to establish a national health data service, the Health Data Research Service (HDRS) [43]. However, given the anticipated multi-year timeline for its implementation, it remains essential to address limitations within the current data access and governance system to enable high-quality, timely research. Doing so is critical for improving health services and for generating the evidence needed to inform the updating of policies and procedures.

## The Road to Hell Winds On

In previous work [8] we reported the challenging and sometimes demoralising burden of setting up our data linkage study LAUNCHES QI: Linking AUdit and National datasets in Congenital HEart Services for Quality Improvement (Box 1). LAUNCHES QI has now ended, 3.5 years later than intended (partly, but definitely not wholly, due to the pandemic). During the project (and after) we found that the administrative burden and associated costs do not end once you have your data.

In this paper we take you further along the road to hell, highlighting the ongoing requirements and challenges from our experience of applying for pseudonymised patient level health data from audits and national datasets in England to ultimately link together to analyse. We uniquely describe the total costs, time, and workload associated with the entire project, including study set-up, annual requirements, and ame-ndments. We also describe our experience of re-using the data-set for another study CHAMPION, Congenital Heart Audit: Measuring Progress In Outcomes Nationally (box 1) (which we wrongly assumed would be more straightforward), and report on new challenges encountered. Finally, we summarise the research made possible with the final linked datasets.

## Total Document Burden

### Set-Up

The full list of documents required for the set-up of both studies is given in Supplementary Table 1 (minor amendments not included in this table). The study set-up for LAUNCHES required 39 documents spanning 345 pages, shared with 11 regulators, with multiple submissions of many of the documents (133 submissions in total). Although re-using the LAUNCHES dataset (plus some additional data), CHAMPION was considered a new study and required the same set-up process as LAUNCHES (described in box 1) with additional documents required for the extra datasets, the addition of online forums, and an increase in document requirements and document size due to changing processes. In total, CHAMPION set-up involved 44 documents spanning 445 pages, shared with 12 regulators, with 143 document submissions in total. For one data controller, CHAMPION was added as an amendment to the LAUNCHES Data Sharing Agreement, which added complexity when amendments were made to one study and not the other.

### Once Studies Are Underway

The statistics above just relate to study set-up. Table 1 shows the documents required throughout the rest of each study for LAUNCHES and CHAMPION.

**Table 1:**
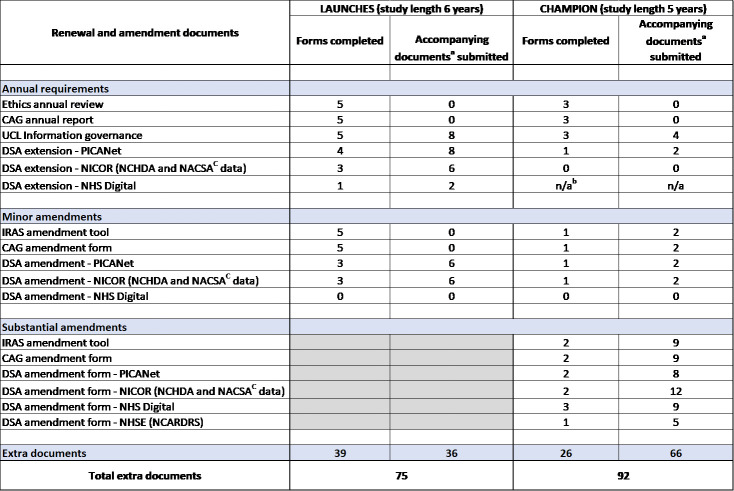
Additional Documents Required Throughout LAUNCHES and CHAMPION

^a^New versions of protocol, data linkage diagram, privacy notice, ethics amendment approval, CAG amendment approval, and in one case the re-sharing of all documents from the original data application.

^b^The CHAMPION NHSD application was submitted as an amendment to the LAUNCHES application, therefore the annual renewal was covered in the LAUNCHES renewal.

^c^NACSA data was for CHAMPION only.

CAG = Confidentiality Advisory Group; DSA = Data Sharing Agreement; UCL = University College London; IRAS = Integrated Research Application System; PICANet = Paediatric Intensive Care Network; NICOR = National Institute for Cardiovascular Outcomes Research; NCHDA = National Congenital Heart Disease Audit; NACSA = National Adult Congenital Surgery Audit; NHSE = NHS England; NCARDRS = National Congenital Anomaly and Rare Disease Registration Service.

Annual requirements involved the maintenance of our approvals: a review for the research ethics committee (REC), a report for the Confidentiality Advisory Group (CAG), information governance updates (university specific), and reviews submitted to most of the data controllers to renew the Data Sharing Agreement (DSA) annually. We note that the projected end dates (several years in the future) were known and shared with all regulators from the start.

As each study progressed and we began working with the datasets, changes and improvements such as the addition of objectives, changing project staff, and extensions to the study end date, needed to be made which required the completion of amendment forms and processes. Combined with the required annual documentation to maintain compliance, this resulted in a considerable ongoing burden throughout each study.

During LAUNCHES we submitted five minor amendments. When combined with annual requirements, 75 documents were added to the study document set over the course of the study, increasing the overall document total for LAUNCHES to 104.

During CHAMPION we submitted three minor and two substantial amendments. We completed an extra 26 amendment/annual renewal forms and submitted 66 study documents to accompany the forms (these included new versions of study documents, and approvals received for the amendments), meaning in total an additional 92 documents for amendments and annual requirements, with a study total of 136. These totals are correct as of March 2025 but will increase as annual requirements will continue for as long as we retain the data. 

**Figure:**
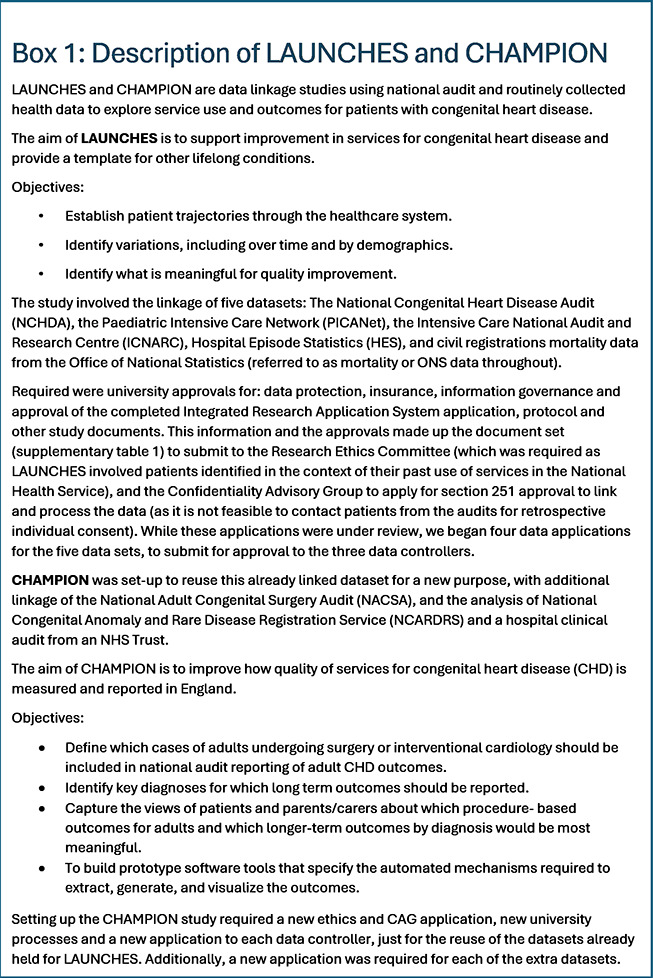


## Total Data Costs

Data linkage studies are expensive, with charges possible from each data processor and controller for application review, data processing, linkage, and transfer costs.

Table 2 shows the total data costs for each dataset for both LAUNCHES and CHAMPION. At the time of reporting, the total data costs for LAUNCHES are £55,650. Following the study end in October 2023, data is being retained to complete analyses and publications. This retention will require annual renewals for some of the data sharing agreements, with associated costs. Therefore, the costs will continue for as long as we hold the data.

**Table 2:**
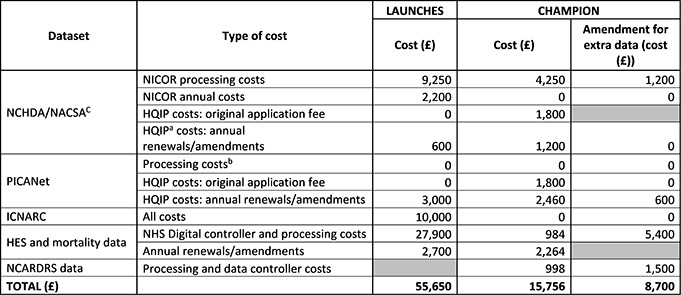
Data Controller and Processor Costs for LAUNCHES and CHAMPION

^a^Data controller changed to NHSE for the amendment for extra data.

^b^PICANet did not charge processing costs at the time of these two studies, but have subsequently introduced charges that will apply to future studies.

^c^NACSA data was for CHAMPION only.

PICANet = Paediatric Intensive Care Network; NICOR = National Institute for Cardiovascular Outcomes Research; NCHDA = National Congenital Heart Disease Audit; NACSA = National Adult Congenital Surgery Audit; HES=Hospital Episode Statisitcs; HQIP = Health Quality Improvement Partnership; NHSE = NHS England; NCARDRS = National Congenital Anomaly and Rare Disease Registration Service.

The cost to re-use the data for CHAMPION, to receive two additional datasets, and for the annual requirements came to £15,756. The substantial amendment that included an update of the NCHDA and ONS data extracts from 2017 to 2022 came to £8,700. The total data costs for CHAMPION to date are £24,456.

## Total Time Taken

**Figure 1:**
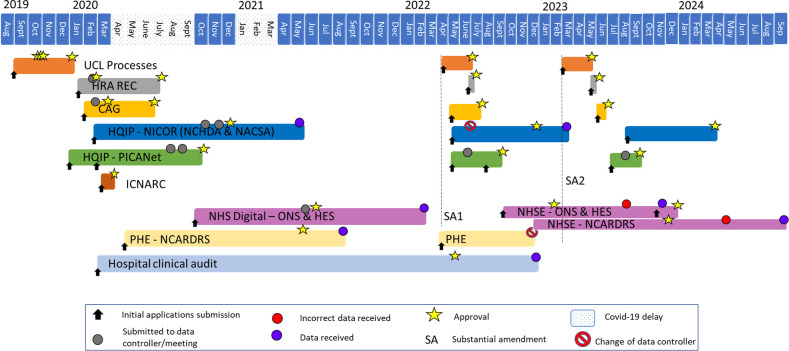
Timeline of the Permission Processes and Time Taken in CHAMPION: From First Submission to Approval and Data received if Applicable, for Each Regulator

Figure 1 shows the timeline for CHAMPION from study set-up and for each substantial amendment. Supplementary Figure 1 (SF1) shows the timeline for LAUNCHES updated from the one previously reported [8].

For LAUNCHES, the time taken to receive approval from each of the regulators ranged from 4 to 9 months. From study start to receiving the final dataset was 2.5 years. Minor amendments (not all shown) took between 1 and 4 months for each of the necessary approvals.

For CHAMPION, permissions to re-use the data we already held at UCL took 1 year and 10 months with individual approval times ranging from 1 to 22 months. It was a further 1 year and 6 months before we had received all additional datasets requested.

During CHAMPION, two substantial amendments were submitted. The first was to add an objective, to request an update of the NCHDA data and the mortality data, and to perform an additional link of the NCARDRS dataset to NCHDA and mortality data. The second amendment was to add an objective that had been flagged as important by stakeholders, to extend the study end date to have time to complete the new objective, and to make changes to data items that we would be receiving for the NCARDRS dataset (we were early users of the dataset and so despite liaising with the data controllers had requested variables that were not fit for purpose in the initial application).

It took ten months to receive all approvals for substantial amendment 1, and a further seven months to receive the NCHDA and mortality data requested. The dataset for NCARDRS data was only received two years later, and with only one month left to go until study funding ended.

The timeline runs from the first submission at the beginning of CHAMPION to the last approval of the second substantial amendment (minor (non-substantial) amendments are not shown).

CAG = Confidentiality Advisory Group, HQIP = Health Quality Improvement Partnership, PICANET = Paediatric Intensive Care Audit Network, ICNARC = Intensive Care National Audit & Research Centre, NICOR = National Institute For Cardiovascular Outcomes Research, HRA REC = Health Research Authority Research Ethics Committee, UCL = University College London, NACSA = National Adult Congenital Surgery Audit, NCARDRS = National Congenital Anomaly and Rare Disease Registration Service, NCHDA = National Congenital Heart Disease Audit, NHSE = NHS England, ONS = Office for National Statistics, HES Hospital Episode Statistics, PHE = Public Health England.

## Process delays

Throughout the studies we encountered major delays specific to both studies and some delays through broader system challenges. With the benefit of our experience from LAUNCHES, we were prepared for the challenges for setting up CHAMPION, or so we thought. Challenges from LAUNCHES, such as uncertainty over the processes and intricacies required of individual data controllers, were not the issue, as we had the experience from LAUNCHES. However, there were new challenges, as set out below.

### Study Specific Delays

#### The Pandemic

Delays due to the pandemic were impossible to anticipate, but it is important to note that this contributed in part to the delays in the approval process, where ethics, CAG, and the data controllers rightly prioritised applications for COVID research, and the funders allowed us to pause the study with only the study set-up continuing. Early communication with the funders has been key for all delays, and thankfully, they were understanding.

#### Data Errors

Three out of nine of our data extracts were missing requested fields or detail. Waiting for the corrected extracts caused a 1 year and 5 months delay, a 4-month delay, and a 5-month delay.

#### Change in data Controller

Two of the data controllers with whom we had data sharing agreements changed during the study. This occurred whilst we were submitting an amendment and there were no processes in place to deal with our request. This resulted in approvals taking six months for one application and a one year wait for the second application, just for approvals. As one new data controller was not familiar with the details of the study, we had to complete the full application form again, and re-share original documents from the initial approvals, as well as provide additional clarifications. The data teams from the original data controllers tried their best to help us push our applications through, but there was just no process in place.

Receiving the data then took an additional 7 months for one data controller, and the second dataset was only received 2 years later, with just one month left on the study. As we would not have been able to complete the analysis planned for this data, we had to request a variation to contract and minor amendments to extend the study end date.

### System challenges

#### The Preparation

Although not a new challenge, the preparation time is relevant to both studies, and an important consideration for anyone thinking of starting a data linkage study. Our timelines (Figure 1 and Supplementary Figure 1) start from submission to the regulators and do not show the huge task of preparing and submitting the set-up documents. It took two months to prepare the document set and complete other aspects of study set-up prior to submission for LAUNCHES, and as CHAMPION was a mixed methods study the preparation time was a little longer.

#### The Increasing Burden

In the year from the study set-up of LAUNCHES in 2018 to the study set-up of CHAMPION in 2019, the number of documents required, the number of questions on application forms, and the number of clarifications required all increased. In 2023 we started a third data linkage study, and the requirements have increased again.

Encouragingly, following the change of data controller from NHS Digital to NHS England in 2022, there were attempts to streamline the DARS system. As these were our lengthiest applications, and the ones requesting the most clarifications, this is a welcome change. As hopeful as we were for that development, a current application for HES data for a third data linkage study has been submitted, there are significant delays due to staff shortages, and we were waiting to be assigned a case officer for 6 months. The reason for this delay was the complexity of our study, which although not greater in complexity than LAUNCHES and CHAMPION, did not fall directly into the new automated system. Now with the DSA approved and signed after working with DARS for 5 months, we received the NHSE data just under one year from submission, with only the bridge files to allow linkage left to follow.

There are other signs of systems growing in complexity. One of our funders is now asking for an additional form from award holders of research programmes to detail data access management plans (DAMPs). They state that in standardising the process they are aiming to reduce the duplication. But this new form *is* the duplication. We can assure funders that the system already has data management covered, in great detail. Perhaps these are useful for studies not requiring ethics and CAG but, in our experience, this was another deadline to meet, received at the worst possible time, during the set-up of the study. The content was mainly covered in our CAG application, and any other questions (e.g. format of data to be received) would be covered when applying for the data.

#### Patient and Public Involvement and Engagement (PPIE)

PPIE is incredibly important and detailed PPIE plans are required in funding applications. For both LAUNCHES and CHAMPION we had a PPIE group to provide feedback and for CHAMPION a PPI co-applicant and three partner charities. In addition to this, CAG requested at review of our application that additional PPIE activities were completed to ascertain that the use of the data for the specific research questions has the support of patients. Although important, PPIE activity is not effective as a tick box exercise. It can be time consuming, requires proper and experienced facilitation, and support from relevant communities. It is difficult to achieve as additional unfunded work, and if done incorrectly could be damaging to a study or to public support.

#### Amendments

During a study, if anything changes from the original application then the amendment process must be completed. Amendments, classified as minor or substantial, increase paperwork and can take several months to be approved. Each amendment requires not only the completion of the amendment form to describe the changes, but a careful re-evaluation of what was written in the original application sometimes written years previously to ensure that the study remains compliant.

Minor amendments included common project events such as adding researchers or extending the study end date and were required for each regulator. Each resulted in a delay of a few months. The irony that delays in data access are often the reason for study extension in the first place is not lost on us. Substantial amendments such as adding objectives, requesting an update of data, or additional linkage caused a significant delay that resulted in the extension of our study end date for CHAMPION.

#### Insanity is Doing the Same Thing Over and Over Again

The repetition of information across multiple forms and documents required for study set-up is repeated for amendments, on several forms with different formats and slightly different wording for ethics, CAG, and each data application. By the second substantial amendment, the absurdity of this really kicks in.

Some data controllers have made amendments a little easier by having a separate amendment form where only the changes need to be completed, but you still need to repeat it for every dataset. However, despite this, on two occasions we were asked to complete a full application form rather than just the amendment form as there was confusion about the background of the study. Where they do not have an amendment form (e.g. NHS Digital now NHSE), we were often re-questioned on aspects of the original study that had already been clarified and approved.

Table 3 shows the extent of the repetition in the application forms. Most of the information provided in the CAG form is then required again for every data application. Unfortunately, it is not just a case of copy and paste, as each question will be slightly different.

**Table 3:**
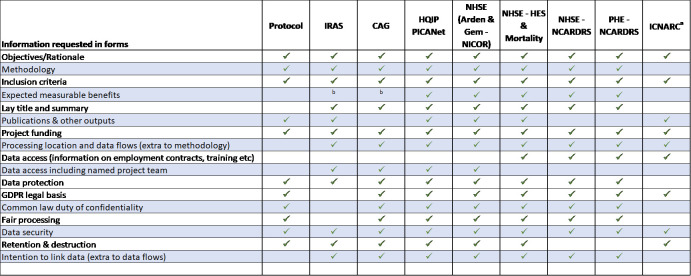
The Repetition of Information Across Application Forms and Documents

Each tick indicates information required in the corresponding form

^a^ICNARC updated their form in March 2024 to include benefits. ^b^Ethics and CAG review the research as a whole to establish that public benefit justifies accessing data.

CAG = Confidentiality Advisory Group; IRAS = Integrated Research Application System; PICANet = Paediatric Intensive Care Network; HQIP = Health Quality Improvement Partnership; NICOR = National Institute for Cardiovascular Outcomes Research; NHSE = NHS England; PHE = Public Health England; NCARDRS = National Congenital Anomaly and Rare Disease Registration Service; HES = Hospital Episode Statistics; ICNARC = Intensive Care National Audit and Research Centre; GDPR = General Data Protection Regulation.

#### It’s Urgent, When it’s Our Move

Renewals and clarifications are chased with urgency when it is our turn to respond but can then sit within the system for months at a time, where the urgency apparently disappears. For example, we waited several months to receive the new process for amending and renewing DSAs following a change of data controller and were told that we would be informed once it was in place. We were not informed, but instead contacted 18 months later to say we were now outside of our current DSA and the renewal was urgent. We completed our side urgently but received the final signed off version 8 Months later.

Some data controllers requested that renewals were submitted up to 3 months before the DSA expiry, but when it’s taken several months to get approval, and then several more months to receive the data, it is frustrating to have to look at it again so soon.

#### The Lonely Burden of the Research Coordinator

Keeping up with changing processes, several data controllers, annual requirements, and the annual expiry of DSAs can become stressful. Research coordinators are doing their best to keep the study compliant and navigate the system but must worry about the smallest deviation from wording written several years earlier being considered a breach of the data sharing agreement. The research coordinator role is crucial and yet often not adequately funded and valued (or remunerated), is needed for the full length of these projects, and ideally full time (at least for the set-up of the project).

#### The Fate of the Dataset

As LAUNCHES has ended and CHAMPION is in its last few months, the data controller’s concern becomes the destruction of the dataset. We have a data retention period for the completion of analyses and publications but after that would be expected to destroy all the data. During data retention, we will need to keep renewing the DSAs which means more documents and costs. Because these fall outside of the funding period, this may not be feasible for the full time planned.

The ultimate destruction of the dataset will be huge waste, given the time, money, and resources used to access, link, clean and prepare the data, as many research questions that could be addressed with these linked datasets, but outside the scope of our studies, remain unanswered. However, the data governance requirements to set up a secure repository where the data could be used by others and updated have already discouraged two potential custodians for the data. Instead, our funder for LAUNCHES provided additional funds to curate the computer code we developed to process the datasets into a public shared repository, which will make it easier for future researchers to recreate and then use linked datasets.

#### Not Sure? Add a Form

The system’s answer to new regulations or oversight is often to add in a form, a question, a clarification, or a process without considering where else this may already be answered or covered. Additions are seen as small administrative tasks, so it’ll be quick. However, whilst it may seem like a small change to an individual data processor or controller, it all adds to the burden. We have found university and NHS R&D processes can be particularly prone to responding in this way.

#### “You Will be in Breach of the DSA if…”

There is a pressure on all parties to get the governance exactly right, but there are grey areas. When we asked questions to ensure we got it right (e.g. whether adding a researcher was allowed without an amendment given that had *optionally* specified the number of researchers accessing the data initially), we often received generic text back, which typically states that any deviation from what is written in the Data Sharing Agreement (DSA) is a breach of that contract, which certainly contributes to *our* fear of legal misstep. Studies change, objectives are added, team members leave and are replaced. It is important that this can happen to allow the best possible research to be carried out. However, any change means that the precise information provided at the beginning of the study might no longer be correct (and so require an amendment). Therefore, from the research team point of view, to navigate the fear of legal misstep the initial application process becomes a trade-off of trying to provide all information and being completely transparent but not providing detail that may later change and make us in breach of the DSA. It is exhausting.

More broadly, it is our belief that the fear of legal misstep within the system is contributing to the burden and delays in accessing data, with each actor behaving as if it is the only regulator and therefore must assess all aspects of the project for itself, despite the study already having been scrutinised by a number of other regulators.

## Advice for Researchers (and Their Employers) Planning a Data Linkage Study

This advice is additional to that offered in Taylor et al [8], and the list is available in Supplementary Table 2. Whilst this advice may help prevent some delays, our experience and learning did not result in a noticeably easier process.

### Keep Submitted Versions of All Applications

Keep all previous versions of submitted applications. Depending on the system, changes may be made to online applications by the data controllers themselves. If these changes are not correct and are missed by the research team, then this could be a breach. For example, one data sharing agreement included the information for both LAUNCHES and CHAMPION. When edits were made by the data controller team, the information for one study had been removed entirely. If we had not picked this up before signing, we would have had one study proceed without the DSA in place. Keeping track of the original request will help if any errors are made.

### Prepare for Extra Costs

Allow for amendments and renewals in data costings. The amendments will normally extend the date of the DSA, however costing in both these and the renewals will provide a buffer should costs change or an amendment be required just after a renewal.

### Study Set Preparation Time

Factoring in suitable research coordinator and PI time at the beginning of the study will aid the preparation of complete and good quality applications. Many decisions are still being made at the start and assuring the wording is correct in the ethics and CAG applications (the first to be submitted) is important to allow consistency across the applications. Any changes made in a later application may cause a cycle of the amendment process. The more complex the study, and the more study methods used, the more preparation time needed.

### Look After the Research Coordinators

They’ve probably just been told they’re in breach of a DSA because of a typo from 3 years ago, whilst carrying on with the general coordination of the study. They can keep on top of the documents, expiry dates of DSAs, and will be able to organise annual requirements and amendments. Having a supportive research team makes all the difference.

## Suggestions for System Improvements – It’s Not Us, It’s You

There will be times when researchers do not know the full requirements or miss new requirements that have just been added. That said, we’ve often received very positive feedback on the quality of our applications (most recently for the extensive ethics application for our latest project). Sadly, we’ve not experienced the process becoming easier or quicker despite the amount of experience we’ve amassed. The delays are so widespread, with many independent examples [1–23], that we are confident in saying that the system is the problem. Ultimately, we believe that the only way to make things significantly less burdensome is to change the system.

**Figure 2:**
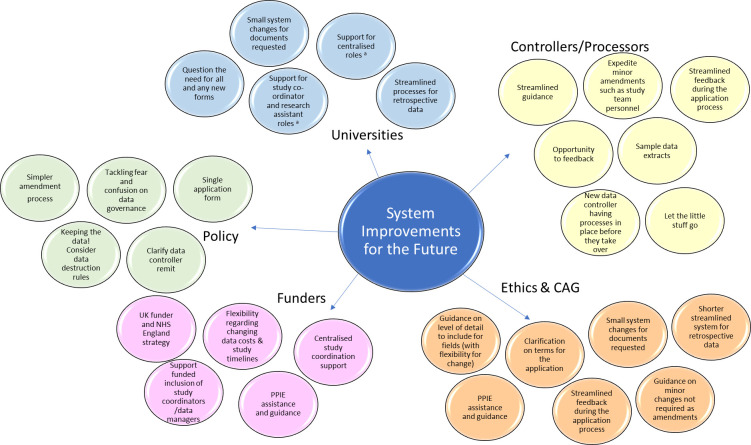
Illustrates Systems Improvements for the Future

Figure 2 is an updated diagram of our suggested system improvements. Additions from Taylor et al [8] include:

The coloured bubbles contain the suggestions, with each colour representing an authority that should consider these changes. CAG, Confidentiality Advisory Group.

^a^Whilst one of our suggested system improvements is for centralised support for study coordinator and research assistant roles, we support the inclusion of these roles to remain within the study team. This is important for teamwork, maintaining enthusiasm for the study aims and study progress, and gives a sense of belonging to staff in a team. All qualities that are being lost as increasing numbers of university professional service roles are centralised, undervalued, and sidelined.

### Sample Data Extracts Before Application

Sample data extracts of requested fields and levels of completeness for these fields, would enable researchers to understand which fields are needed, as well as make sure that the data requested is suitable for the analyses planned. It may also help to trouble shoot any misunderstanding about derived fields (which are particularly important for data minimisation) before the extract has been finalised and so reduce additional work for the data processor in arranging a corrected extract and avoid delays to the data recipient.

### New Data Controllers to have Processes in Place Before Taking on New Datasets

New data controllers should have processes in place before they take over. Studies have been left in limbo without a process, wasting time and funding.

### PPIE Assistance

If the integration of the PPIE into the study design that is currently in place is not seen as adequate for CAG and data law requirements, then centralised support from CAG in accessing PPIE participants, groups, and facilitation would be needed.

Such support would also be useful from funders, as finding and securing required numbers of PPIE participants that are able to commit to the full study time, feel properly included and heard and incorporating their opinions and experiences is a large task.

### Partnership Working Between Funders and Data Providers

Greater partnership between research funders and data providers at a strategic and operational level remains important to ensure aligned support of health data linkage studies.

### Single Application Form (and a Simpler Amendment Process)

We previously suggested a single application form and process for data applications, and after several amendments this seems even more pertinent. This is not only a time burden at the beginning of the study, but minor amendments that need to be completed on 5+ forms become a substantial piece of work, and a substantial amendment becomes an epic one. Even if a single application form is not feasible, there is so much overlap between forms (Table 2) that the same structured questions would surely be possible, with each controller being able to add their own agreement terms. The CAG form is the first to be completed, and the most comprehensive. If these questions could be used in data application forms, then it would allow the same answers (that would still be relevant and correct), to be used.

### Let the Little Stuff Go

Greater flexibility would reduce the need for amendments, saving time for both data controllers, processors, and study teams. For example, having to list the team members when a study can go on for years makes amendments inevitable. Not all data controllers request this information but may still want to know the number of people accessing the data. Even allowing an estimated maximum number of researchers, and so be flexible to small changes, would be useful. That way, a study team hasn’t immediately set themselves up for a breach when they hire someone 2 years later.

Another example is requiring specific grammar. Some forms are required to be written in the first person, some in the third person. Surely this doesn’t matter and often prevents the re-use of already prepared and relevant answers. Small system changes could make a big difference.

A few of the forms required for ethics (e.g. the Organisational Information Document) may not be relevant to the study, but you still must complete the first section, select that this form is not applicable to you, obtain authorisations from the university, and submit it. Getting your head round lengthy forms that are not necessary whilst trying to arrange the other 30 or so forms you do need, is not helpful. Often the lengthy approval process will not begin until all forms, including those not applicable, are submitted.

Most data controllers provide feedback once, requesting any clarifications, which if made well, means the application can proceed. However, some will request clarifications over several months and your application may bounce repeatedly and quickly back to you for small reasons, which can be really disheartening.

## Keep Going - the Value of Linked Datasets

Conscious that we do not want to deter researchers from data linkage studies, we would like to say that although it was incredibly challenging and morale-sapping, we did get there, were able to complete the study objectives, and completed analyses that would not have been possible without linking the data.

For the LAUNCHES objectives we have published eight papers to date with five in preparation, and from CHAMPION we have published nine papers, with two in preparation. We have reported the resource use, outcomes, and difference in hospital burden for several congenital heart disease diagnoses [44–47] and reported for the first time the differences between hospitals when children transfer to adult services [48], and will be reporting on how the adult CHD population use services. We have also looked at long term outcomes by CHD diagnosis [49], and developed risk models for use in routine monitoring of 30-day and 90-day survival in adults undergoing surgery for CHD and for 30-day complications following CHD surgery in children and in adults [50–52]. Using the delayed NCARDRS dataset we evaluated outcomes for all detected structural CHD cases in England from fetal life to the age of 1 year, and reported on the under estimation of cases due to no intervention [53].

## Conclusion

With increasing recognition of the challenges of using health data for research and our own increasing experience at navigating the system, we were hoping for positive change in the 3.5 years since Road to Hell was published. Instead, the forms have proliferated, and processes have become harder, not easier. We believe that simple changes could greatly streamline the system, and yet with each new study we undertake (we are currently doing this process for a new project), the document set increases, and the application forms, the steps to approval and the delays get longer. Doing research with linked datasets is incredibly painful and there is a big disincentive to make amendments which stifles innovation. We have been able to realise the research value of the linked dataset, but the current system delays research and many researchers might just give up without simpler processes to routinely link and update data.

## Supplementary Files

Supplementary Appendices

## Data Availability

The research dataset is not available for sharing under the current data sharing agreements.

## Abbreviations

CAG:	Confidentiality Advisory Group
DSA:	Data Sharing Agreement
GDPR:	General Data Protection Regulation
HES:	Hospital Episode Statistics
HRA:	Health Research Authority
HQIP:	Health Quality Improvement Partnership
ICNARC:	Intensive Care National Audit and Research Centre
IG:	Information Governance
IRAS:	Integrated Research Application System
JRO:	Joint Research Office
NACSA:	National Adult Congenital Surgery Audit
NCARDRS:	National Congenital Anomaly and Rare Disease Registration Service
NCHDA:	National Congenital Heart Disease Audit
NHSE:	NHS England
NICOR:	National Institute for Cardiovascular Outcomes Research
PHE:	Public Health England
PICANet:	Paediatric Intensive Care Network
UCL:	University College London
