# Supplementary material for: The Road to Hell Winds On: The High Administrative Burden of Maintaining Linked National Health Data
Source: Int J Popul Data Sci. 2026 Apr 30;11(1):3000. doi: 10.23889/ijpds.v11i1.3000 (PMC13216846; doi:10.23889/ijpds.v11i1.3000)
Supplement: Supplementary Appendices [file ijpds-11-3000-s001.pdf]

Supplementary appendices

Supplementary Table 1: Document set for study set-up for LAUNCHES and CHAMPION

	Regulator												LAUNCHES		CHAMPION	
	Data protection	Information Governance	JRO office	HRA REC	CAG	Named NHS Site	NHS site of 1 collaborator	HQIP NICOR	HQIP PICANet	ICNARC	NHS Digital	Public Health England	Pages/ screens	Times submitted	Pages/ screens	Times submitted
**Study forms filled out**																
Data Protection form													7	3	8	3
**Governance forms (online)**													1	1	5	1
IRAS form													32	4	37	4
**CAG form – IRAS**													40	5	42	5
CAT advice form													5	1	0	0
**HQIP form (NICOR)**													29	4	20	2
HQIP form (PICANet)													24	2	20	2
**ICNARC form**													5	2	5	2
DARS form (online)													21	2	20	2
PHE form for NCARDRS													0	0	15	2
NHS DSA for Barts clinical audit													0	0	12	2
**Data fields & justification (NICOR)**													4	1	4	1
Data fields & justification (PICANet)													4	1	4	1
**Data fields & justification (ICNARC)**													4	1	0	0
**Data fields & Justification (NCARDRS)**													0	0	8	1
Data Linkage Diagram													1	9	0	0
**Detailed data linkage diagram**													1	1	1	9
Risk Assessment													3	3	2	1
**IG toolkit confirmation**													1	8	0	0
Correspondence with NICOR													1	2	0	0
**Correspondence with charities**													1	1	0	0
Letter from SIRO													1	3	1	3
**Privacy notice**													2	9	4	10
**Privacy notice - short description**													0	0	1	10
Protocol													25	6	14	6
**Insurance registration form**													6	1	14	1
Statement of Activities													22	4	0	0
**Organisational Info Document**													7	4	10	4
**Schedule of events**													3	4	3	4
Cover Letters													3	3	2	3
**Checklist**													2	2	2	2
**DPIA**													0	0	3	4
**Signature & delegation log**													0	0	3	2
**Patient information**													0	0	21	4
**PPI opinions on data use**													0	0	2	1
**Documents organised/shared**																
**HQIP letter of support**													1	3	1	1
Insurance Certificate													1	1	1	1
**REC provisional opinion**													5	2	6	2
REC favourable opinion													4	8	6	8
**CAG provisional approval**													8	4	10	4
CAG approval													6	8	9	8
**Funder Contract**													38	2	52	2
PI CV													4	4	2	4
CV's from team													10	1	4	1
Reviewers Comments													4	4	2	4
HRA/HCRW approval letter													4	7	6	8
Training certificates													5	2	6	2
Service agreement													0	0	14	3
Draft collaboration agreement													0	0	37	2
Honorary contracts													0	0	6	1
**Totals**													345	133	445	143

Key:

- Form required for both LAUNCHES and CHAMPION
- Form required for LAUNCHES only
- Form required for CHAMPION only

Block shading indicates documents required for each corresponding regulator. The 'times submitted' column sums the number of times each document was required by a different regulator. *Data protection, Information Governance and JRO processes as at UCL.

IRAS = Integrated Research Application System, CAG = Confidentiality Advisory Group, CAT = Confidentiality Advice Team, PICANET = Paediatric Intensive Care Audit Network, ICNARC = Intensive Care National Audit & Research Centre, DARS = Data Access Request Service, NICOR = National Institute For Cardiovascular Outcomes Research, IG = Information Governance, SIRO = Senior Information Risk Owner, HQIP = Health Quality Improvement Partnership, HRA REC = Health Research Authority Research Ethics Committee, PI = Principal Investigator, CV = Curriculum Vitae, HCRW = Health And Care Research Wales, UCL = University College London, JRO = Joint Research Office.

Supplementary Table 2: Previously reported advice for your data linkage studies [8]

**Advice**	**Explanation**
Find people who have been there before you	Researchers who were further ahead in their data application journey shared their learning and expertise with us. This helped speed up the initial process of preparing the applications.
Love thy research assistants/study coordinators (the legal amount)	These are roles that often get squeezed at application stage to reduce costs and can be challenging to resource if part-time. But these roles are absolutely crucial in accessing data.
Have a flexible start up period	Having an initial data application period in the project with just the PIs and a study coordinator funded allowed us to react early to delays and quickly adjust the timeline.
Add clinical and audit experts to the team	Input from clinicians on data fields helped with data minimisation versus research realisation (particularly a problem for exploratory work). We also found it useful to include, where possible, collaborators working for data controllers and audits.
Plan your data minimisation and beware of differences in how some terms are interpreted	You will need to describe how you have minimised the need for patient identifiers, and all fields requested will require justification. In doing so be sure to correctly state if data will be de-identified, pseudonymised or further anonymised. Some consider anonymised data to be aggregate data only, and amendments may be needed to clarify terms.

PI=Principal Investigator.

Supplementary Figure 1: Updated Timeline of the Permission Process Order and Time Taken in LAUNCHES QI: From First Submission to Data Acquisition

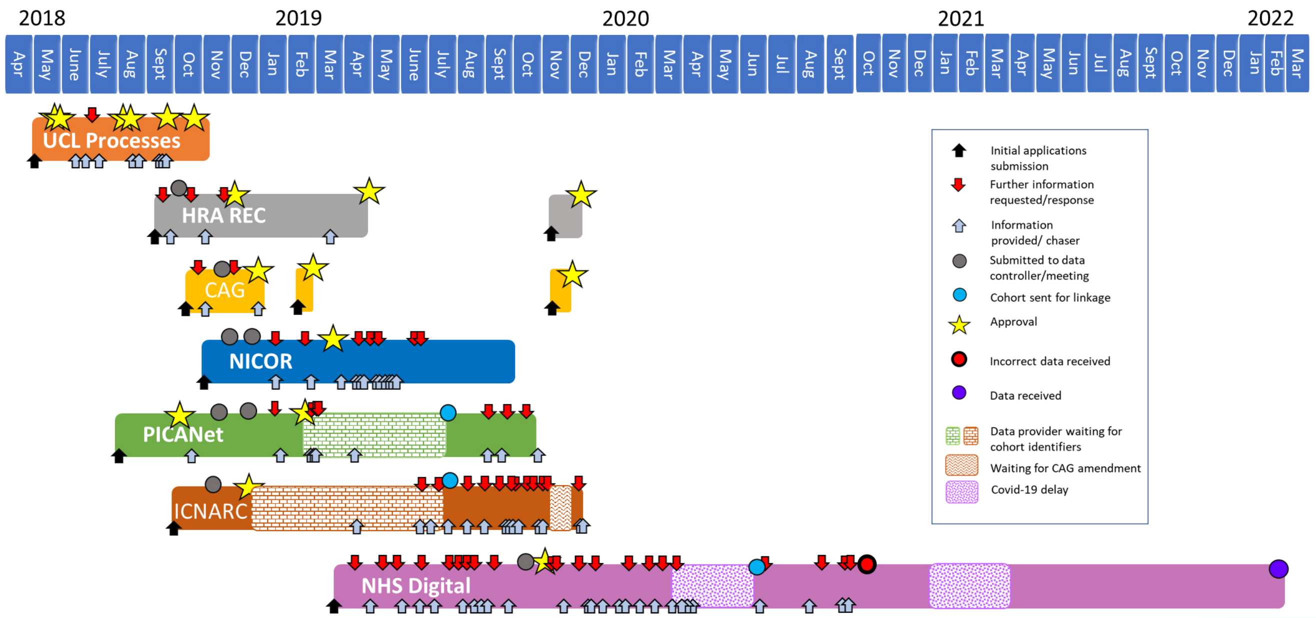

Each box represents the time from submission of the data application to transfer of the data to UCL, for each data set. Note that not all requests for further information and responses have been included! CAG, Confidentiality Advisory Group; HRA, Health Research Authority; ICNARC, Intensive Care National Audit and Research Centre; NHS, National Health Service; NICOR, National Institute for Cardiovascular Outcomes Research; PICANet, Paediatric Intensive Care Audit Network; REC, Research Ethics Committee; UCL, University College London.
